# Medial meniscus posterior root tear with concomitant focal cartilage lesion may be successfully treated with pullout repair combined with additional bone marrow stimulation

**DOI:** 10.1016/j.asmart.2026.05.012

**Published:** 2026-06-09

**Authors:** Yuki Okazaki, Yusuke Yokoyama, Masanori Tamura, Koki Kawada, Kazuhisa Sugiu, Tsubasa Hasegawa, Toshiki Kohara, Toshifumi Ozaki, Takayuki Furumatsu

**Affiliations:** a Department of Orthopaedic Surgery, Okayama University Graduate School of Medicine, Dentistry, and Pharmaceutical Sciences, 2-5-1 Shikata-cho, Kita-ku, Okayama City, Okayama, 700-8558, Japan; b Department of Orthopaedic Surgery, Okayama Red Cross Hospital, 2-1-1 Aoe, Kita-ku, Okayama City, Okayama, 700-8607, Japan

**Keywords:** Arthroscopy, Bone marrow stimulation, Cartilage lesion, Clinical outcome, Medial meniscus, Posterior root tear

## Abstract

**Background/objective:**

Although many studies have reported the clinical outcomes of pullout repair for medial meniscus posterior root tears (MMPRTs) or microfractures for focal cartilage lesions alone, no study has specifically reported the clinical outcomes in patients with MMPRT combined with cartilage lesions. Therefore, we investigated the effectiveness of bone marrow stimulation via microfracture for treating focal cartilage lesions associated with MMPRT and compared the clinical outcomes between patients who underwent microfracture and those who did not. We hypothesised that favourable clinical outcomes could be achieved after transtibial pullout repair combined with microfracture for MMPRTs with focal cartilage lesions and that these outcomes would be comparable to those after transtibial pullout repair alone for MMPRTs without cartilage lesions.

**Methods:**

Ten patients who underwent transtibial pullout repair and microfracture for MMPRT with cartilage lesions (Group MF) and 37 patients who underwent only pullout repair for MMPRT without cartilage lesions (Group P) using the same pullout technique (two simple stitches) as Group MF between November 2016 and September 2023 were retrospectively evaluated. The age at the time of surgery, sex, height, weight, body mass index, duration from injury to surgery, and KL grade were recorded for each patient. The presence, location, and area of cartilage lesions were evaluated. Clinical outcomes were assessed, and all included patients underwent a second-look arthroscopy.

**Results:**

The mean follow-up period, patient age, body mass index, femorotibial angle, and percentage of the mechanical axis in Group MF were 3.2 years, 60.2 years, 26.1 kg/m^2^, 176.3°, and 42.4%, respectively. All postoperative clinical outcomes at one and three years showed significant improvement compared with preoperative outcomes, including the KOOS and IKDC, Tegner, Lysholm, and pain VAS scores. Second-look arthroscopy confirmed good meniscal healing. No significant differences in demographic data, radiological parameters, clinical outcomes, or meniscal healing were observed between Groups MF and P, both preoperatively and at the three-year postoperative follow-up.

**Conclusion:**

The clinical outcomes of transtibial pullout repair combined with microfracture were favourable in patients with MMPRT and focal cartilage lesions in well-aligned knees. Moreover, no significant differences in clinical outcomes were detected between patients who underwent pullout repair combined with microfracture and those who underwent pullout repair alone. MMPRTs with focal cartilage lesions may be effectively treated with pullout repair combined with microfracture, provided that strict patient selection criteria are applied.

## 1 Introduction

Medial meniscus (MM) posterior root tears (PRTs) disrupt hoop stress distribution, leading to rapid progression of cartilage damage,^1^ subchondral insufficiency fractures, and osteoarthritis.^2^^,^^3^ These lead to abnormal tibiofemoral joint biomechanics, MM posteromedial extrusion during knee flexion, and overloading of the articular cartilage due to an inability to convert axial loads into hoop stresses.^4^, ^5^, ^6^ Although a high body mass index and varus knee alignment are independent risk factors for this condition, MM posterior root repair is currently the recommended treatment and is preferred over conservative therapies or partial meniscectomies for MMPRTs based on biomechanical and long-term follow-up clinical studies.^7^, ^8^, ^9^

Varus knee alignment contributes to poor short-, medium-, and long-term postoperative clinical outcomes in patients with MMPRTs. A varus alignment of >5° is the principal risk factor for this condition.^10^, ^11^, ^12^, ^13^ Therefore, MMPRT with moderate-to-severe varus knee deformities is typically treated with high tibial osteotomy, either independently or in combination with MMPRT repair^14^^,^^15^ or unicompartmental knee arthroplasty.^16^ Although the optimal alignment (percentage of the mechanical axis, femorotibial angle [FTA], and hip-knee-ankle angle) for pullout repair in MMPRT remains controversial, a zero-to-mild varus deformity is often considered a good indication for pullout repair alone, with reports of favourable mid- to long-term clinical outcomes.^8^^,^^17^ However, the clinical outcomes and indications for knees with zero-to-mild varus alignment and focal cartilage lesions have not yet been reported. Nevertheless, cases of MMPRT with cartilage lesions can occur.

Various treatment options are available for cartilage defects.^18^ Surgical treatment of focal chondral lesions involves bone marrow stimulation. The lesion area, measured as width × length (cm^2^), is typically determined after debridement. The location and size of the defects are estimated preoperatively using magnetic resonance imaging and definitively assessed during arthroscopy. Many studies have confirmed that microfractures result in better clinical outcomes in the treatment of smaller defects (2–4 cm^2^) in younger patients.^19^ Despite many reports on the clinical outcomes of pullout repair for MMPRT or microfractures for focal cartilage lesions alone,^18^ no study has specifically reported the clinical outcomes in patients with MMPRT combined with cartilage lesions. Therefore, in this study, we aimed to investigate the effectiveness of bone marrow stimulation using the microfracture technique for treating focal cartilage lesions associated with MMPRT in well-aligned knees. We also compared the clinical outcomes with those of patients who did not undergo microfracture. We hypothesised that favourable clinical outcomes could be achieved after transtibial pullout repair combined with microfracture for MMPRTs with focal cartilage lesions and that these outcomes would be comparable to those after transtibial pullout repair alone for MMPRTs without cartilage lesions.

## 2 Materials and methods

### 2.1 Patients

This study was approved by the Institutional Review Board (#1857) and conducted in accordance with the principles of the Declaration of Helsinki. Written informed consent was obtained from all participants prior to participation. Initially, 12 consecutive patients who underwent transtibial pullout repair and microfracture for MMPRT associated with cartilage lesions (Group MF) and 40 patients who underwent only pullout repair (Group P) using the same pullout technique (two simple stitches, TSS) as Group MF between November 2016 and September 2023 were enrolled.

The inclusion criteria were as follows: 1) arthroscopic transtibial pullout repairs of MMPRT; 2) femorotibial angle (FTA) < 180°; 3) Kellgren–Lawrence (KL) grade of 0–2; and 4) focal cartilage lesions (≤1.5 cm^2^), including severe International Cartilage Repair Society (ICRS) grade (grades Ⅲ and Ⅳ). Although FTA was used as the eligibility criterion, the % mechanical axis was additionally assessed to provide a comprehensive radiographic evaluation of lower limb alignment. The ≤1.5 cm^2^ threshold reflects our institutional indication for microfracture,^18^ which is reserved for small, focal defects, whereas larger lesions are treated with alternative cartilage restoration procedures. Patients who did not undergo second-look arthroscopy (one patient in each group) or who had severe varus deformity (FTA ≥180°) were excluded from the analysis. Ultimately, 10 patients in Group MF and 37 patients in Group P were included in this study.

Age at the time of surgery, sex, height, weight, body mass index, duration from injury to surgery, and KL grade were recorded for each patient. The injury date was defined as the date when patients reported experiencing a “painful posteromedial popping episode”.^20^ MMPRT types were classified based on tear morphology, as previously described for types 1–5.^21^ Cartilage lesions were also evaluated arthroscopically. The meniscal healing status was evaluated by a senior orthopaedic surgeon during second-look arthroscopy, one year after arthroscopic transtibial pullout repair. Healing was assessed using a previously published semiquantitative arthroscopic scoring system described by Furumatsu et al.,^22^ which was developed to evaluate meniscal healing after MMPRT repair and demonstrated clinical relevance through a significant correlation with the quality of life subscale of the Knee injury and Osteoarthritis Outcome Score (KOOS). The semiquantitative scoring system was as follows. 1) Anteroposterior width of the bridging tissues between the MM posterior horn and root attachment: broad/narrow/filamentous scored as 4/2/0, respectively. 2) Stability of the repaired MM posterior root: good/fair/loose/useless/detached scored as 4/3/2/1/0, respectively. 3) Synovial coverage of the sutures: good/fair/poor scored as 2/1/0, respectively. Meniscal healing was scored on a scale of 0–10 points.^22^

### 2.2 Magnetic resonance imaging measurement

Imaging was performed using an Achieva 1.5 T scanner (Philips, Amsterdam, The Netherlands) or EXCELART VantageTM powered by Atlas 1.5 T with an integrated coil (Toshiba Medical Systems, Tochigi, Japan). Standard sequences included sagittal/coronal (TR/TE 5000/107) T2-weighted fat suppression with a 90° flip angle. The slice thickness was 4 mm with a 0.6-mm gap, and the field of view was 16 × 16 cm, with an acquisition matrix size of 512 × 410, or 3 mm, with a 0.6-mm gap, and the field of view was 18 cm with an acquisition matrix size of 224 ×320.

The presence, location, and area of the cartilage lesions were evaluated. The area of the cartilage lesion (cm^2^) was calculated by approximating it as an ellipse using the anteroposterior (AP, in the sagittal plane), transverse (TR, in the coronal plane), and craniocaudal (CC, in the sagittal plane) dimensions. The formula was as follows: area = 1/4 × (π × AP × TR for lesions in the medial femoral condyle [MFC] and medial tibial plateau [MTP]) or 1/4 × (π × TR × CC for lesions in the trochlea).

### 2.3 Surgical technique and rehabilitation protocol

Patients with MMPRTs underwent arthroscopic transtibial pullout repair and postoperative rehabilitation, as previously described.^23^^,^^24^ Briefly, TSS was used to grasp the posterior horn and root. Tibial fixation of the sutures was performed using a double-spike plate (Meira, Aichi, Japan) or a bioabsorbable screw (Smith & Nephew, London, UK) at 20°–30° knee flexion with an initial tension of 20–30 N. Subsequently, the patients were initially kept non-weight-bearing with a knee immobiliser for two weeks. Between two and four weeks, knee flexion exercises gradually increased under partial weight-bearing conditions. After five or six weeks, the patients were permitted full weight-bearing and knee flexion of 120°.

### 2.4 Clinical outcomes

Clinical evaluations were performed at the time of arthroscopic transtibial pullout repair for MMPRT and at one, three, and five years postoperatively. The following clinical outcomes were assessed: Lysholm score (0 = worst, 100 = best), Tegner activity score (0 = worst, 10 = best), International Knee Documentation Committee (IKDC) score (0 = worst, 100 = best), pain visual analogue scale (VAS) (0 = no pain, 100 = worst possible pain), and KOOS, which comprises five subscales (pain, symptoms, activities of daily living, sports and recreational function [Sport/Rec], and knee-related quality of life).

### 2.5 Statistical analysis

Data are presented as mean ± standard deviation. Statistical analyses and power calculations were performed using EZR software (Saitama Medical Centre, Jichi Medical University, Tochigi, Japan). For longitudinal intragroup comparisons, a linear mixed-effects model was applied with time as a fixed effect and patient as a random effect to account for within-subject correlation and missing data. Post hoc pairwise comparisons were performed using the Bonferroni adjustment. Intergroup differences were compared using the Mann–Whitney U test, and categorical variables were analysed using Fisher's exact test or the chi-square test, as appropriate. Statistical significance was set at *p* < 0.05.

## 3 Results

The mean follow-up period for Group MF was 3.2 years. Table 1 presents the patient characteristics at the time of surgery in both groups. In Group MF, longitudinal analysis using a linear mixed-effects model demonstrated a significant overall effect of time for all clinical outcomes, including KOOS subscales, IKDC, Tegner, Lysholm, and pain VAS scores (all *p* < 0.05, Table 2). Post hoc comparisons revealed significant improvements from the preoperative baseline to one and three years follow-up. Five-year outcomes were available for only three patients; therefore, these data are presented descriptively.

**Table 1:** Preoperative patient demographics.

	Group MF	Group P	*p*-value
Number of patients	10	37	
Sex, male/female (number)	3/7	7/30	0.42
Age (years)	60.2 ± 7.2	64.0 ± 7.1	0.11
Height (m)	1.58 ± 0.09	1.56 ± 0.06	0.62
Weight (kg)	65.8 ± 12.0	61.4 ± 11.2	0.42
Body mass index (kg/m^2^)	26.1 ± 3.4	25.1 ± 3.4	0.38
Root tear classification, type 1/2/4	1/9/0	3/33/1	0.86
Duration from injury to surgery (weeks, n = 9 and 29, respectively)	13.2 ± 10.5	7.1 ± 5.7	0.07
Duration from surgery to final follow-up (years)	3.2 ± 1.5	3.0	
Severe cartilage damage (trochlea/trochlea + MTP/MFC)	2/1/7	N/A	

Data are presented as the number or mean ± standard deviation or number (range). MFC, medial femoral condyle; MTP, medial tibial plateau; N/A, not applicable; MF, pullout repair combined with microfracture; P, only pullout repair.

**Table 2:** Longitudinal changes in clinical scores following medial meniscus posterior root repair combined with microfracture (Group MF).

	Preoperative	One-year (n = 10)	Three-year (n = 8)	Five-year (n = 3)
KOOS				
Pain	54.9 ± 24.1	84.1 ± 11.0	83.8 ± 19.0	95.4 ± 5.8
Symptoms	56.8 ± 23.7	80.1 ± 9.4	89.2 ± 11.9	91.6 ± 8.3
ADL	60.0 ± 21.1	86.9 ± 8.1	88.9 ± 12.0	94.6 ± 6.0
Sport/Rec	29.4 ± 30.2	56.5 ± 21.5	64.2 ± 24.0	71.7 ± 12.6
QOL	32.2 ± 23.1	60.7 ± 12.5	64.7 ± 21.2	70.8 ± 7.2
IKDC score	35.4 ± 18.3	60.2 ± 10.2	68.8 ± 17.7	75.5 ± 1.3
Lysholm score	57.7 ± 6.7	84.5 ± 9.4	91.7 ± 4.6	90.5 ± 4.2
Tegner activity score	1.3 ± 0.7	3.3 ± 1.2	3.5 ± 1.2	3.3 ± 0.6
Pain visual analogue scale	41.6 ± 32.2	18.6 ± 21.8	13.3 ± 25.5	2.7 ± 3.1

Values are presented as mean ± standard deviation. Longitudinal changes were analysed using a linear mixed-effects model with time as a fixed effect and patient as a random effect. Significant overall time effects were observed for all outcomes (all*p* < 0.05). Five-year data are presented descriptively due to the limited sample size. ADL, activities of daily living; IKDC, International Knee Documentation Committee; KOOS, Knee Injury and Osteoarthritis Outcome Score; QOL, knee-related quality of life; Sport/Rec, sports and recreational function; MF, pullout repair combined with microfracture.

**Fig. 1:**
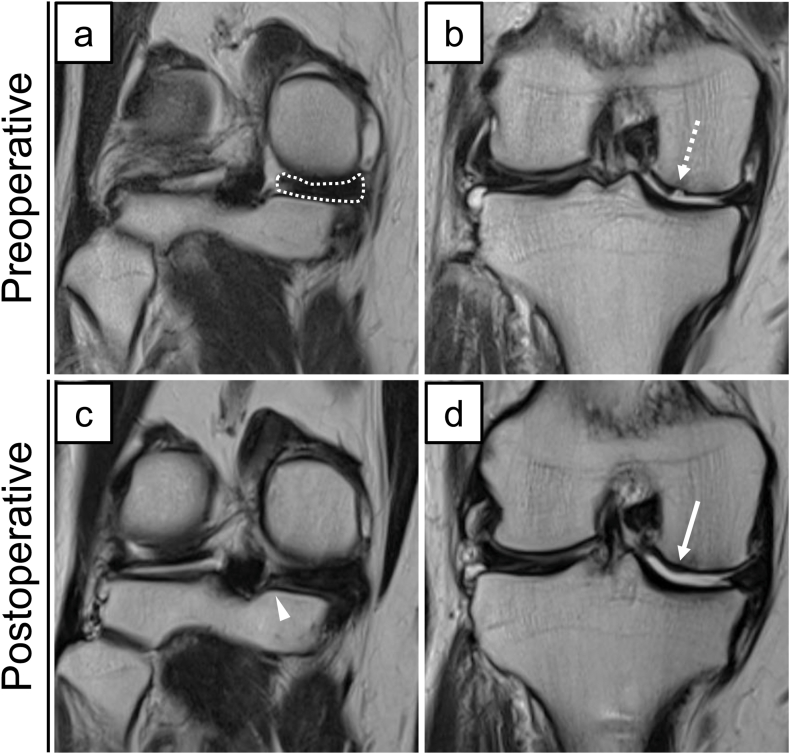
Representative magnetic resonance images of the right knee undergoing pullout repair for medial meniscus (MM) posterior root tear (PRT) combined with microfracture. a) Preoperative coronal image showing giraffe neck sign (white dotted area), indicative of MMPRT. b) Preoperative coronal image showing a cartilage lesion on the medial femoral condyle (MFC). c) Postoperative coronal image showing restored continuity of the MM posterior root. d) Postoperative coronal image showing improvement in the cartilage lesion on the MFC.

**Fig. 2:**
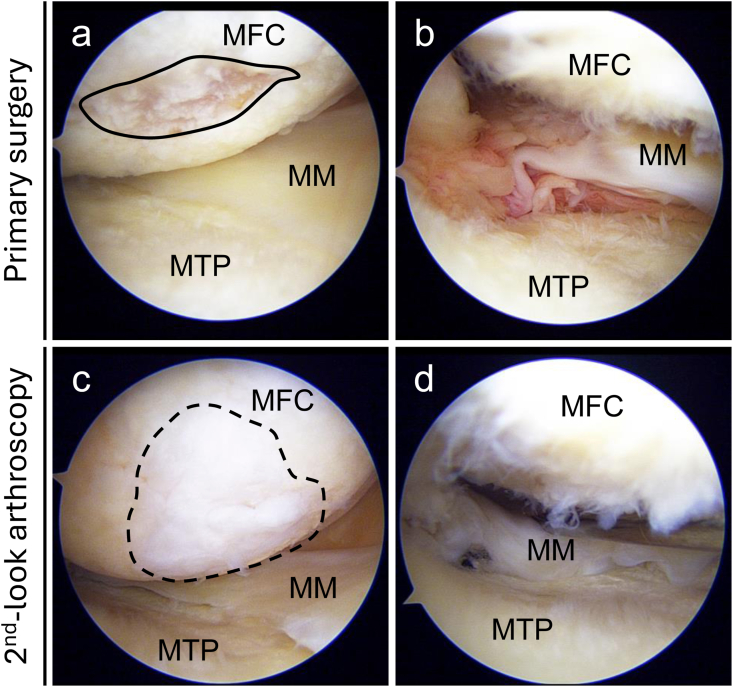
Arthroscopic findings from primary surgery (a, b) and second-look arthroscopy (c, d) in patients with medial meniscus (MM) posterior root tear (PRT) concomitant with severe cartilage damage on the medial femoral condyle (MFC). a) Microfracture performed for severe cartilage damage (International Cartilage Repair Society [ICRS] grade 4) on the MFC (solid area). b) MMPRT confirmed during the procedure. c) Fibrocartilage coverage observed on the MFC during second-look arthroscopy, with ICRS grade improvement from 4 to 2 (dashed area). d) Confirmed healing of the MM.

**Fig. 3:**
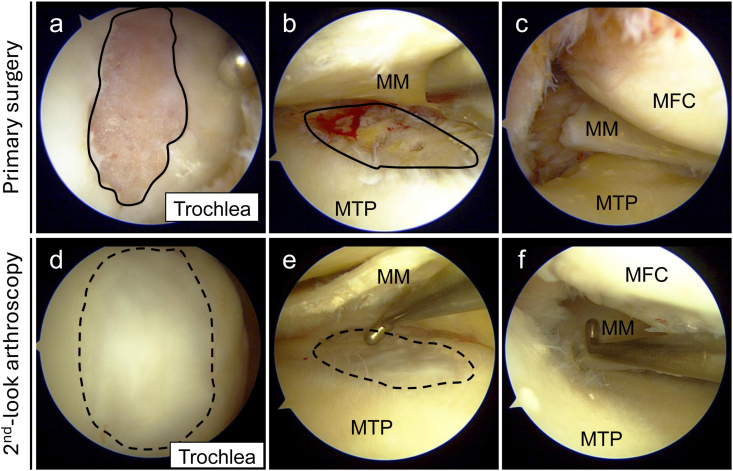
Arthroscopic findings from primary surgery (a–c) and second-look arthroscopy (d–f) in patients with medial meniscus (MM) posterior root tear (PRT) concomitant with severe cartilage damage on the trochlea and medial tibial plateau (MTP). a) Microfracture performed for severe cartilage damage (International Cartilage Repair Society [ICRS] grade 4) on the trochlea (solid area). b) Microfracture performed for severe cartilage damage (ICRS grade 4) on the MTP (solid area). c) MMPRT confirmed during the procedure. d) Fibrocartilage coverage observed on the trochlea, with ICRS grade improvement from 4 to 2 (dashed area). e) Fibrocartilage coverage observed on the MTP, with ICRS grade improvement from 4 to 2 (dashed area). f) Confirmed healing of the MM.

The ICRS grade improved in all patients, regardless of whether the affected area was a loading (MFC [Fig. 1, Fig. 2] and MTP [Fig. 3]) or unloading (trochlea [Fig. 3]) zone (Table 3). No significant differences were observed in the preoperative clinical scores, radiological parameters (Table 4), or three-year postoperative outcomes between Groups MF and P (Table 5).

**Table 3:** Comparison between preoperative and postoperative values following medial meniscus posterior root repair combined with microfracture (Group MF).

	Preoperative	Postoperative
ICRS grade (trochlea, n = 3; 0/1/2/3/4)	0/0/0/1/2	0/0/2/0/0
ICRS grade (medial tibial plateau, n = 1; 0/1/2/3/4)	0/0/0/0/1	0/0/1/0/0
ICRS grade (medial femoral condyle, n = 7; 0/1/2/3/4)	0/0/0/2/5	0/0/7/0/0
Medial meniscus extrusion (mm)	4.3 ± 0.9	5.4 ± 1.2
Kellgren–Lawrence grade (0/1/2/3)	0/1/9/0	0/0/5/5
Arthroscopic healing score	N/A	7.7 ± 1.3

ICRS grade and medial meniscus extrusion are assessed using one-year postoperative arthroscopy and magnetic resonance imaging, respectively. The Kellgren–Lawrence grade is determined via plain radiographs at one year in two cases, at three years in five cases, and at five years in three cases. ICRS, International Cartilage Repair Society; MF, pullout repair combined with microfracture.

**Table 4:** Comparison between preoperative clinical scores and radiological parameters between Groups MF and P.

	Group MF	Group P	*p*-value
KOOS			
Pain	54.9 ± 24.1	57.1 ± 18.2	0.72
Symptoms	56.8 ± 23.7	57.7 ± 20.8	0.91
ADL	60.0 ± 21.1	63.9 ± 19.7	0.37
Sport/Rec	29.4 ± 30.2	24.1 ± 24.4	0.81
QOL	32.2 ± 23.1	38.3 ± 22.0	0.45
IKDC score	35.4 ± 18.3	46.3 ± 26.8	0.86
Lysholm score	57.7 ± 6.7	60.6 ± 13.9	0.51
Tegner activity score	1.3 ± 0.7	1.8 ± 0.9	0.13
Pain visual analogue scale	41.6 ± 32.2	46.3 ± 26.8	0.85
Femorotibial angle (°)	176.3 ± 1.7	176.8 ± 1.9	0.91
% mechanical axis (%)	42.4 ± 8.1	40.6 ± 6.9	0.88
Kellgren–Lawrence (grade 1/2)	1/9	12/25	0.24
Medial meniscus extrusion (mm)	4.3 ± 0.9	4.0 ± 1.1	0.49

Values are presented as mean ± standard deviation. MF, pullout repair combined with microfracture; P, only pullout repair; ADL, activities of daily living; IKDC, International Knee Documentation Committee; KOOS, Knee Injury and Osteoarthritis Outcome Score; QOL, knee-related quality of life.

**Table 5:** Comparison of three-year postoperative clinical scores, radiological parameters, and arthroscopic score between Groups MF and P.

	Group MF	Group P	*p*-value
KOOS			
Pain	83.8 ± 19.0	86.9 ± 9.9	0.68
Symptoms	89.2 ± 11.9	82.2 ± 15.0	0.15
ADL	88.9 ± 12.0	85.8 ± 10.6	0.18
Sport/Rec	64.2 ± 24.0	51.9 ± 30.0	0.24
QOL	64.7 ± 21.2	63.9 ± 22.4	0.91
IKDC score	68.8 ± 17.7	62.8 ± 17.2	0.36
Lysholm score	91.7 ± 4.6	85.2 ± 10.9	0.32
Tegner activity score	3.5 ± 1.2	2.9 ± 0.7	0.21
Pain visual analogue scale	13.3 ± 25.5	13.8 ± 14.4	0.36
Medial meniscus extrusion (mm)	5.4 ± 1.2	5.4 ± 1.6	0.91
Kellgren–Lawrence grade (1/2/3)	0/7/3	1/26/13	0.82
Arthroscopic healing score	7.7 ± 1.3	7.5 ± 1.2	0.80

Values are presented as mean ± standard deviation. The data in Group MF are from eight patients who were followed for three years or more. MF, pullout repair combined with microfracture; P, only pullout repair; ADL, activities of daily living; IKDC, International Knee Documentation Committee; KOOS, Knee Injury and Osteoarthritis Outcome Score; QOL, knee-related quality of life.

## 4 Discussion

In this study, we demonstrated that transtibial pullout repair combined with microfracture resulted in favourable clinical outcomes in patients with MMPRT and focal cartilage lesions in well-aligned knees and that no significant differences were detected in clinical or radiological outcomes compared with patients who underwent only pullout repair without cartilage lesions. The findings partially support our hypothesis. Previous research has reported favourable long-term outcomes with transtibial pullout repair compared with conservative treatment.^8^^,^^17^ Our study also showed that good clinical results might be achieved without the need for osteotomy or osteochondral autologous transfer system when treating mild varus knees with cartilage damage of ≤1.5 cm^2^.

Articular cartilage is avascular and aneural and lacks lymphatic vessels, resulting in poor healing potential. Damaged surfaces that rub against each other accelerate the softening and cracking of cartilage.^25^^,^^26^ Moreover, articular cartilage lesions have poor repair capacity, leading to progressive joint damage.^18^ Conservative treatment involves physical therapy and intra-articular injections. Intra-articular injections of hyaluronic acid and platelet-rich plasma promote healing of cartilage injuries and improve the lubricating properties of the joint.^27^ However, these treatment methods are not widely accepted, especially for young patients, and have limitations in patients with large cartilage defects and significant angular deformities of the knee joint.^28^ In such situations, minimally invasive techniques based on bone marrow stimulation or the use of osteochondral grafts should be considered.^18^ In this study, cartilage lesions were limited to ≤1.5 cm^2^. Although microfracture has historically been applied to defects up to 2–4 cm^2^, recent clinical practice trends favor more restrictive indications, generally recommending microfracture for lesions smaller than approximately 2–2.5 cm^2^.^18^ The ≤1.5 cm^2^ threshold reflects our institutional treatment strategy, in which larger defects are treated with alternative restorative procedures, such as osteochondral autologous transplantation. Furthermore, microfracture was limited to small, focal defects (≤1.5 cm^2^). The limited size of the lesions may have reduced the biological demand for defect filling and mechanical resilience, potentially allowing satisfactory fibrocartilage repair even under a rehabilitation protocol not specifically tailored for cartilage restoration. Therefore, the present findings may not be generalizable to larger cartilage defects, in which postoperative mechanical loading conditions could exert a more substantial influence on repair tissue quality.

The knee joint comprises the patellofemoral and tibiofemoral joints, which differ substantially in terms of pressure generated during movement. Rehabilitation after surgery should consider these biomechanical differences, with particular attention to the location and extent of the injury, as well as the surgical technique used. In this study, the rehabilitation protocol was primarily designed to protect the repaired meniscal root and included an initial, protected weight-bearing phase. Although this protocol was not specifically optimized for cartilage restoration, early restriction of loading may have provided a sufficiently stable biological environment for fibrocartilage formation. Nevertheless, because the rehabilitation strategy was not cartilage-specific, it remains unclear whether an optimized postoperative protocol would have further improved structural or clinical outcomes.

Bone marrow stimulation techniques are widely used in the arthroscopic treatment of cartilage and osteochondral defects. Among these, microfracture, drilling including nanofracture,^29^^,^^30^ and abrasion of the subchondral layer should be considered. These therapies aim to induce the extravasation of bone marrow rich in stromal cells and secondary formation of a fibrin clot, which serves as the biological basis for repair tissue formation, predominantly fibrocartilage rather than hyaline cartilage.^31^ Although animal studies have shown that drilling does not destroy more osteocytes than microfracture,^32^ it can cause thermal damage to the osteocytes and excessive damage to the subchondral layer, resulting in necrosis, hypertrophy, or the development of intraosseous cysts.^33^ Nanofracture, developed as a minimally invasive technique, is a variation of drilling that differs by its smaller hole size (up to 1 mm) and deeper penetration (up to 9 mm).^29^ The advantage of this method is the possibility of a denser distribution of drillings and lesser damage to the subchondral layer at the defect sites. Additionally, osteocytes are not thermally damaged during the procedure. Moreover, nanofracture achieves better healing and anatomical reconstruction of the trabecular bone and less frequent subchondral cyst formation.^34^ The effectiveness of nanofracture has previously been reported, and it could represent an option for femoral condyles. However, to ensure consistent evaluation of the effects of the procedure throughout the study, we adopted the standard microfracture technique, the effectiveness of which has been widely reported in all cases.

Bone marrow cells can differentiate into fibrochondrocytes, resulting in the formation of fibrous cartilage. Fibrous cartilage predominantly comprises type I collagen and exhibits weaker biochemical and biomechanical properties than hyaline cartilage.^35^ Biological augmentation is occasionally considered, as it can lead to significant improvements in patient-reported outcome measures.^36^, ^37^, ^38^ However, this improvement did not reach a minimal clinically important difference (MCID) in any reported trial, and it is concluded that further research is needed to confirm the potential of platelet-rich plasma augmentation to microfracture for the treatment of cartilage lesions.^39^ Therefore, we did not adopt additional biological augmentation, as bone tunnel creation during MMPRT repair may provide access to bone marrow. However, this biological effect was not directly assessed in this study and should be considered hypothetical. Furthermore, restoration of the contact area and pressure in the medial knee compartment following pullout repair^4^^,^^5^ may create an environment conducive to cartilage healing. Patellofemoral cartilage degeneration may progress after pullout repair^40^, ^41^; however, in this study, trochlear microfracture led to cartilage regeneration, albeit as fibrocartilage. A favourable clinical outcome was observed in patients treated with additional microfracture; however, the specific contribution of marrow stimulation cannot be isolated.

This study has some limitations. First, because group allocation was based on the presence or absence of focal cartilage lesions, the effect of microfracture cannot be completely separated from differences in baseline cartilage status. Therefore, this study does not allow for a definitive evaluation of the independent effect of microfracture itself. Rather, the findings should be interpreted as a comparison of clinical outcomes between patients with MMPRT and cartilage lesions treated with additional microfracture and those without cartilage lesions treated with pullout repair alone. In addition, second-look arthroscopy was not performed in one patient in each group due to patient preference. Although the number was small and balanced between groups, the requirement for second-look arthroscopy may introduce selection bias, as patients declining re-arthroscopy may differ clinically from those included in the analysis. Second, the sample size was small. Although no *a priori* power analysis was performed due to the retrospective design, the observed intergroup differences did not exceed previously reported MCID thresholds for the IKDC score (10.9) or KOOS subscales (11.4–16.9).^42^ Therefore, although the possibility of a Type II error cannot be completely excluded, the observed intergroup differences were small and did not exceed established MCID thresholds for most outcomes. Third, histological and biomechanical analyses were not performed, leaving the composition and stiffness of fibrocartilage unknown, which may necessitate further investigations, such as animal experiments. Finally, the postoperative follow-up period may have been short to fully evaluate the clinical outcomes and subsequent knee-related complications following pullout repair and microfracture in patients with MMPRT. Furthermore, although a linear mixed-effects model was used to account for repeated measures and missing data, the relatively small sample size, particularly at 5-year follow-up, may limit the robustness of long-term estimates. Accordingly, the present study should not be interpreted as evidence of the isolated efficacy of microfracture. Rather, it evaluates whether acceptable short- to mid-term clinical outcomes may be achieved in carefully selected patients with MMPRT and focal cartilage lesions when microfracture is added to pullout repair.

## 5 Conclusion

The combination of transtibial pullout repair and microfracture resulted in favourable clinical outcomes in patients with MMPRT and focal cartilage lesions in well-aligned knees. Moreover, no significant differences in clinical outcomes were detected between patients who underwent pullout repair with microfracture and those who underwent pullout repair alone. Thus, MMPRT with focal cartilage lesions may be effectively treated with pullout repair combined with microfracture, provided that strict patient selection criteria are adhered to.

## Declaration of interest

The authors have no conflicts of interest relevant to this article.

## Declaration of generative AI and AI-assisted technologies in the manuscript preparation process

During the preparation of this work, the authors used ChatGPT to assist with English-language proofreading. After using this tool/service, the manuscript was professionally proofread by Editage, and the authors subsequently reviewed and edited the content as needed and take full responsibility for the content of the published article.

## Patient consent

Written informed consent was obtained from all patients.

## Ethics approval

This study was conducted in accordance with the principles of the Declaration of Helsinki. The study was approved by the Ethics Committee of Okayama University (No. 1857).

## Funding

This work was supported by 10.13039/501100001691JSPS KAKENHI (grant number 24K23295 and 25K19973).

## Data Availability

Data that support the findings of this study are available from the corresponding author upon reasonable request.
